# ﻿Two new species of the genus *Sinonychus* (Coleoptera, Elmidae) from Guizhou, China

**DOI:** 10.3897/zookeys.1223.122412

**Published:** 2025-01-06

**Authors:** Ri-Xin Jiang, Xiang-Sheng Chen

**Affiliations:** 1 Institute of Entomology, Guizhou University, Guiyang 550025, Guizhou, China; 2 The Provincial Special Key Laboratory for Development and Utilization of Insect Resources of Guizhou, Guizhou University, Guiyang, 550025, Guizhou, China; 3 The Provincial Key Laboratory for Agricultural Pest Management of Mountainous Region, Guiyang 550025, Guizhou, China

**Keywords:** China, Guizhou, identification key, Macronychini, new records, riffle beetles, taxonomy

## Abstract

The riffle beetle genus *Sinonychus* Jäch & Boukal, 1995 (Elminae, Macronychini) includes only three species from East Asia. In this paper, two new species, *S.lipinae***sp. nov.** and *S.luodianensis***sp. nov.**, are described from Guizhou Province, China. Illustrations of the new species and a key to all five *Sinonychus* species are also provided.

## ﻿Introduction

The genus *Sinonychus* Jäch & Boukal, 1995 (Elmidae, Elminae, Macronychini) includes three species from East Asia (Jäch and Boukal 1995; Yoshitomi and Nakajima 2007, 2012). The genus was erected by monotypy with *Sinonychuslantau* Jäch & Boukal, 1995 from Hong Kong, China, the type species. Subsequently, two species from Japan, *S.satoi* Yoshitomi & Nakajima, 2007 and *S.tsujunensis* Yoshitomi & Nakajima, 2012 were added (Yoshitomi and Nakajima 2007, 2012). Kamite and Moriya (2011) recorded *S.satoi* from Amami-Ȏshima, Japan. Hayashi (2019) described the minute structure of the body surface of *S.tsujunensis* adults. Moreover, Hayashi and Kamite (2020) described the immature stages of *S.tsujunensis*.

Members of the genus *Sinonychus* are characterized by the following: 1) body very small, usually less than 1.50 mm; 2) antenna 7-segmented; 3) pronotum wider than long; 4) sublateral grooves present on the pronotum; 5) longitudinal impression of the pronotum extending from its base almost to the anterior margin; 6) elytra obovate, disc more or less roof-like in cross-section; 7) elytral intervals 3, 5, 6, 7 or 5, 6, 7 with carinae; 8) hind wings absent; and 9) legs moderately long (Jäch and Boukal 1995; Yoshitomi and Nakajima 2007).

In this paper, two new species, *S.lipinae* sp. nov. and *S.luodianensis* sp. nov., are described and illustrated from Guizhou Province, China. A key to all five species of *Sinonychus* is provided.

## ﻿Material and methods

Examined material is deposited in the Institute of Entomology, Guizhou University, Guiyang, China (**GUGC**).

Label data of the specimens is quoted verbatim. The Chinese translation of each locality below the provincial level is included in parentheses at the first appearance in the text. Each specimen from the type series bears one of the following labels: “HOLOTYPE (red) (or PARATYPE (yellow)), ♂ (or ♀), *Sinonychus* + specific name sp. nov., Jiang & Chen, 2024.”

Habitus images were taken using a Canon 5D SR camera in conjunction with a Mitutoyo Plan NIR 5 lens. Images of the morphological details were taken using the same camera in conjunction with a Mitutoyo Plan NIR 10 lens or a Nikon DS-Ri2 camera with a Nikon SMZ25 stereoscopic microscope. Zerene Stacker (version 1.04) was used for image stacking. All images were modified and grouped into plates in Adobe Photoshop CS5 Extended.

The following abbreviations are used in the text: **HW**—width of head across eyes; **PL**—length of pronotum along midline; **PW**—maximum width of pronotum; **EL**—length of elytra along suture; **EW**—maximum width of elytra; **CL**—sum of PL + EL.

## ﻿Taxonomy

### ﻿ Sinonychus

Taxon classificationAnimaliaColeopteraElmidae

Jäch & Boukal, 1995

7AF6E832-EDC4-55A0-953C-3CEEB9986FC4

Sinonychus Jäch & Boukal, 1995: 306.

#### Type species.

*Sinonychuslantau* Jäch & Boukal, 1995.

### ﻿ Sinonychus lipinae

Taxon classificationAnimaliaColeopteraElmidae

Jiang & Chen sp. nov.

065EB3C4-CACF-5F1C-A4C8-F5EE1B92DA22

https://zoobank.org/26A5072B-6E7F-4EC1-B403-CD5189584B94

**Figure 1.**
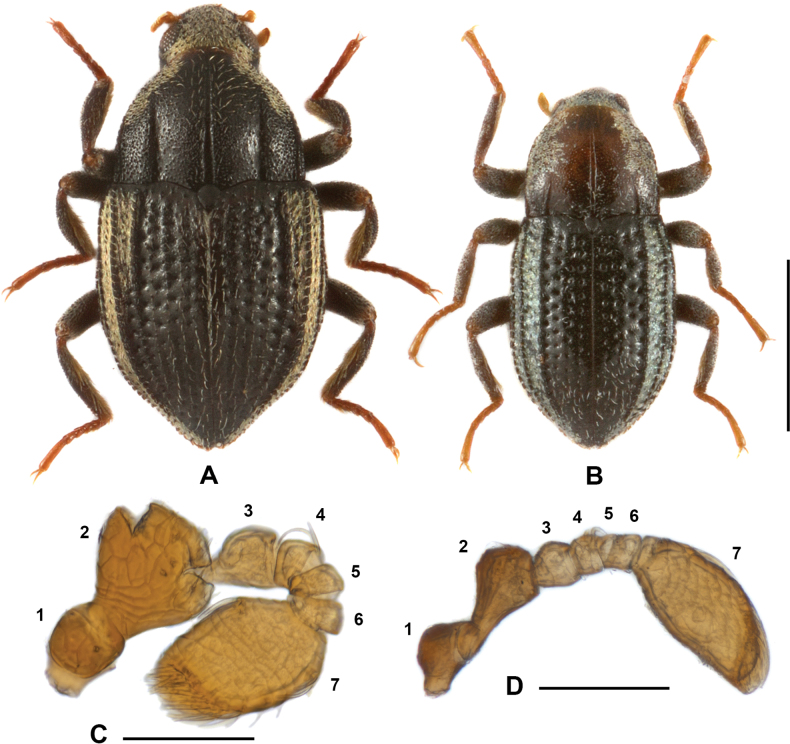
**A** dorsal habitus of *Sinonychuslipinae* sp. nov. **B** dorsal habitus of *S.luodianensis* sp. nov. **C** antenna of *S.lipinae* sp. nov. **D** antenna of *S.luodianensis* sp. nov. Scale bars: 0.5 mm (**A, B**); 0.05 mm (**C, D**).

**Figure 2.**
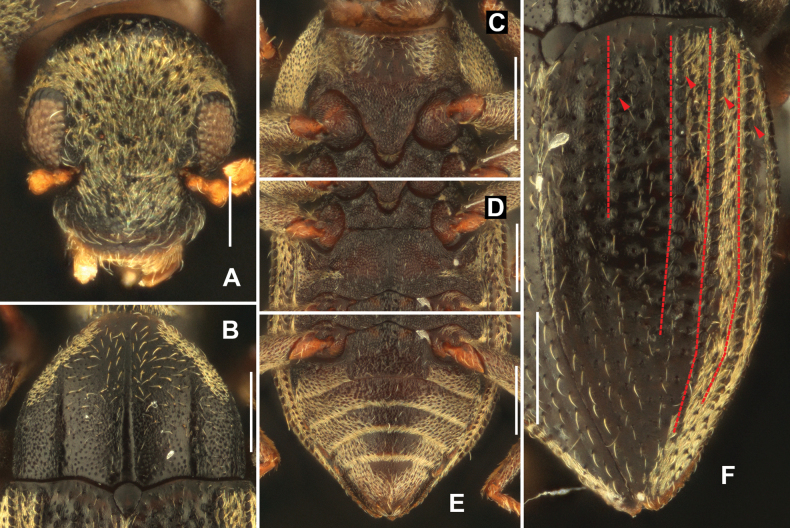
Diagnostic features of *Sinonychuslipinae* sp. nov. **A** head **B** pronotum **C** prosternal process **D** metaventrite **E** abdomen **F** elytron. Scale bars: 0.1 mm (**A**); 0.2 mm (**B–F**).

Figs 1A, C , 2A–F , 4A–G (李氏华溪泥甲)

#### Type material.

26 exs: 11 ♂♂, 5 ♀♀, 10 exs., sex undetermined. ***Holotype***: • China: ♂, labeled “China: Guizhou, Qiannan Buyi and Miao Autonomous Prefecture (黔南布依族苗族自治州), Longli (龙里县), Wantanhe Town (湾滩河镇), H: 1136.10±1.08m, 26°12'52"N 106°59'27"E, 31.VIII.2023, Jiang Ri-Xin, Hai-Tao Li, Pin Li, Yu-Hao Zhang, Yin-Lin Mu & Xiu-Dong Huang leg.” (GUGC). ***Paratypes***: • 10 ♂♂, 5 ♀♀, 10 exs., sex undetermined, with same label data as the holotype (GUGC).

#### Diagnosis.

Body broadly oval, black; mouthparts, antennae, anterior margin of pronotum, trochanters and base of tibia and tarsi (including claws) light brown. Frons, pronotum and basal elytra finely granulate. Pronotum with anterolateral marginal band of silvery, sericeous tomentum. Elytral intervals 3, 5, 6, 7 with granulate carinae; carina of interval 3 short, less than half the length of elytron; other carinae longer than half length of elytron. Aedeagus slender, apex of median lobe acute; median lobe with a pair of long sclerotizations located at apical 1/2.

#### Description.

Body broadly oval (Fig. 1A); black, with mouthparts, antennae, anterior margin of pronotum, trochanters and base of tibia and tarsi (including claws) light brown. Plastron setae confined to the following areas: head (both dorsal and ventral surfaces, including clypeus); pronotum (anterolateral marginal areas); elytra (lateral areas, including epipleura); prosternum (except disc); mesoventrite, metaventrite and abdomen (lateral areas); and femora.

Head (Fig. 2A) wider than long, surface covered with plastron setae and mixed with sparse, long setae and granules. Clypeus anterior surface microreticulate; covered with sparse, long setae; without plastron setae. Labrum transverse, narrower than clypeus; surface microreticulate, apical 1/2 covered with sparse, long setae; apical margin nearly straight; lateral margins rounded and with long bristles. Antenna (Fig. 1C) 7-segmented with apical antennomere clubbed.

Pronotum (Fig. 2B) wider than long, widest at base, get narrowed from basal 1/3 to apex. Surface microreticulate and granulate except areas near apical margin. Apicolateral margins covered with plastron setae. Anterior 1/2 of disc covered with sparse, long setae distinctly longer than setae on other parts of pronotum. Median longitudinal sulcus distinct and long, extending from base nearly to anterior margin, deep at basal 1/2, much shallower at apical 1/2. Sublateral grooves distinct and straight, parallel to each other. Anterior margin strongly curved, anterior angles not produced. Lateral margins nearly parallel at base, then evenly narrowed. Basal margin trisinuate, emarginate anterior to scutellum, posterior angles nearly orthogonal. Prosternal process (Fig. 2C) subtriangular, with rounded apex, surface distinctly microreticulate, covered with sparse, long setae.

Scutellum (Fig. 2B) cordate, longer than wide, widest at basal 1/3; surface shiny and glabrous. Anterior margin strongly curved, lateral margins weakly curved, apex acutangular.

Elytra (Fig. 2F) longer than wide, widest near middle. Surface granulate near base and apex; disc microreticulate. Strial punctures large in basal 2/3 of elytra, mostly separated by about twice a diameter; much smaller and widely separated in other parts of elytra. Elytral intervals 3, 5, 6, 7 with granulate carinae; carina of interval 3 shortest, about 1/3 length of elytra; carina of interval 5 about 2/3 length of elytra; other two carinae long, extending from base of elytra nearly to apex. Areas from interval 5 to lateral margins with plastron setae except for apical 2/3 between intervals 5 and 6. Hing wings reduced.

Metaventrite (Fig. 2D) with disc distinctly microreticulate and covered with sparse, long setae; lateral areas with plastron setae. Median sulcus shallow and indistinct, extending from posterior margin to anterior margin.

Abdominal surface finely granulate (Fig. 2E). Admedian carinae of ventrite 1 obscure, straight, extending from base to apex. Median areas of ventrites 1–4 and anterior middle part of ventrite 5 distinctly microreticulate; lateral areas of ventrites 1–5 covered with plastron setae and mixed with sparse long setae.

Legs simple, surface granulate (except tarsi). Surface of femora covered with sericeous tomentum; inner side of tibiae with cleaning fringes; tarsi slightly shorter tibiae; tarsal claws simple.

Aedeagus (Fig. 4A–D) slender and elongate. Parameres short, not obvious, weakly sclerotized, without setae. Median lobe symmetrical, distinctly narrowed near base; apex acute; with a pair of long sclerotizations at apical 1/2. Sternite IX (Fig. 4E) with apical margin curved, without setae, median strut with base distinctly curved. Phallobase short, about 1/6 length of median lobe.

Measurements: CL: 1.25–1.43 mm; PL: 0.42–0.50 mm, PW: 0.58–0.63 mm; EL: 0.81–0.93 mm, EW: 0.70–0.75 mm.

Female externally similar to the male, but averaging larger. Ovipositor as in Fig. 4F, G: valvifer about twice as long as coxite, distinctly expanded at base; coxite apex strongly expanded, broadly rounded at outer margin; stylus short, distinctly curved at middle.

Measurements: CL: 1.28–1.41 mm; PL: 0.43–0.48 mm, PW: 0.61–0.66 mm; EL: 0.85–0.93 mm, EW: 0.73–0.81 mm.

#### Distribution.

China. Only known from the type locality in Longli County, Qiannan Buyi and Miao Autonomous Prefecture, Guizhou Province.

#### Biology.

All adults were collected from gravel on the bottom of a small stream in a ravine (Fig. 5A–E).

#### Etymology.

The species epithet “*lipinae*” honors our friend and colleague Dr Pin Li (Guizhou University), one of the collectors of this new species.

#### Comparative diagnosis.

*Sinonychuslipinae* sp. nov. is highly similar to the Japanese species *S.tsujunensis* in appearance. The new species can be distinguished from the latter species by the following characters: 1) median longitudinal sulcus of pronotum narrower; basal 1/2 distinctly wider than apical 1/2 (vs. much wider; basal 1/2 weakly wider than apical 1/2); 2) male aedeagus with apex of median lobe acute (vs. apex rounded); 3) parameres without setae in basal parts (vs. bearing short setae in basal parts); and 4) median lobe about 6 times as long as phallobase (vs. about 5 times as long as phallobase).

### ﻿ Sinonychus luodianensis

Taxon classificationAnimaliaColeopteraElmidae

Jiang & Chen sp. nov.

C0ED14C2-30FD-5FCA-94F9-7B2C9B797812

https://zoobank.org/EC1B7D2E-24F8-4320-82A4-3F50B1E6D07C

**Figure 3.**
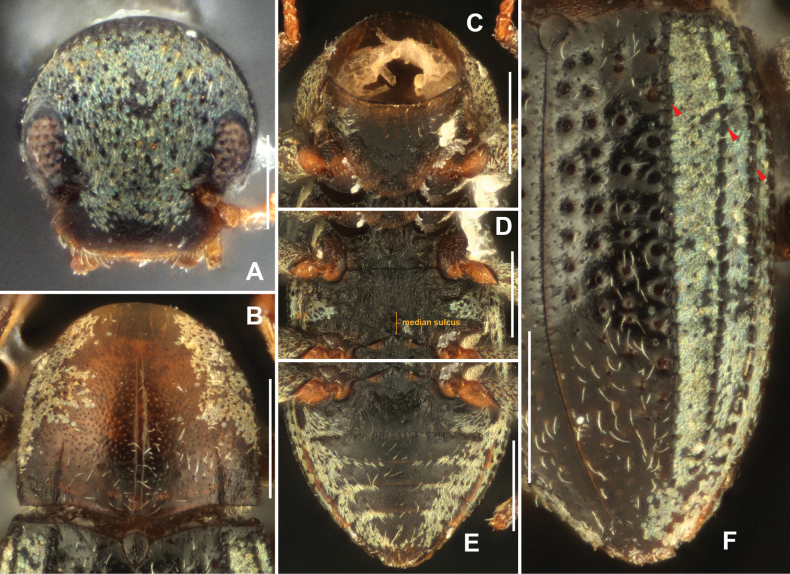
Diagnostic features of *Sinonychusluodianensis* sp. nov. **A** head **B** pronotum **C** prosternal process **D** metaventrite **E** abdomen **F** elytron. Scale bars: 0.1 mm (**A**); 0.2 mm (**B–F**).

**Figure 4.**
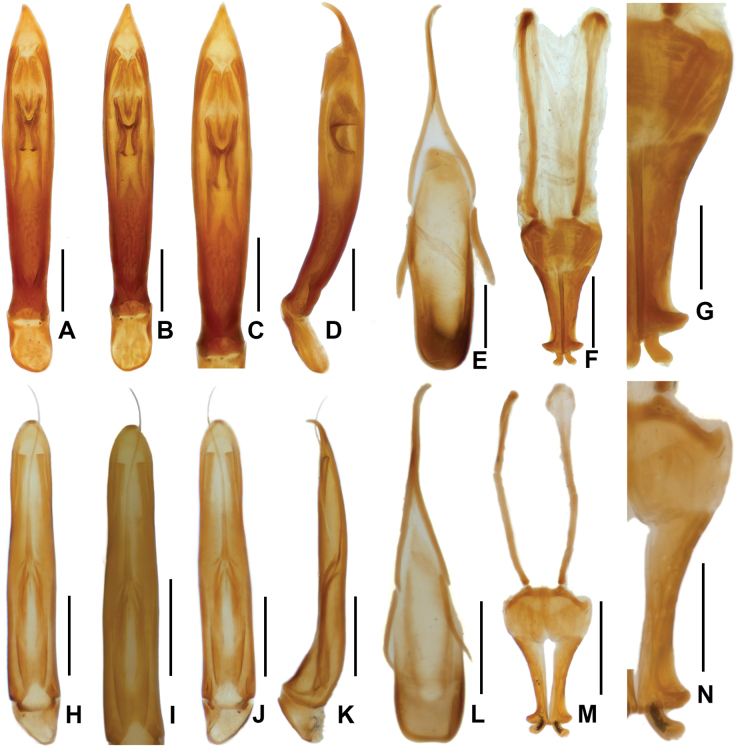
Genital features of *Sinonychus* species: **A–G***Sinonychuslipinae* sp. nov. **H–N**, *Sinonychusluodianensis* sp. nov. **A, H** aedeagus, dorsal view **B, J** ditto, ventral **C, I** ditto, median lobe **D, K** ditto lateral view **E, L** sternite IX **F, M** ovipositor **G, N** ditto, apical part. Scale bars: 0.05 mm (**G, N**); 0.1 mm (**A–F, H–M**).

Figs 1B, D , 3A–F , 4H–N (罗甸华溪泥甲)

#### Type material.

41 exs: 11 ♂♂, 10 ♀♀, 20 exs., sex undetermined. ***Holotype***: • CHINA: ♂, labeled “China: Guizhou, Qiannan Buyi and Miao Autonomous Prefecture (黔南布依族苗族自治州), Luodian County (罗甸县), Luokun Town (罗悃镇), Xiangshui Village (响水村), 25°19'43"N 106°38'28"E, H: 666.10±6.40m, 09.XI.2022, Jiang Ri-Xin leg.” (GUGC). ***Paratypes***: • 10 ♂♂, 10 ♀♀, 20 exs., sex undetermined, with same label data as the holotype (GUGC).

#### Diagnosis.

Body long-oval, mostly black; pronotum (except basal part), antennae, base of tibia and tarsi (including claws) light brown. Plastron setae scaly. Elytral intervals 5, 6 and 7 with granulate carinae. Aedeagus with apex of median lobe rounded; median lobe with two pairs of long, elongate sclerotizations located at basal 1/2; a very thin and long sclerotization extends from middle of median lobe to well beyond the apex, where it is curved.

#### Description.

Body long-oval (Fig. 1B); black with pronotum (except basal part), antennae, base of tibia and tarsi (including claws) light brown. Plastron setae confined to following areas: head (both dorsal and ventral surfaces, including disc of clypeus); pronotum (lateral areas); elytra (lateral areas, including epipleura); prosternum, mesoventrite, metaventrite and abdomen (lateral areas); and femora.

Head (Fig. 3A), wider than long, surface covered with plastron setae and sparse, long setae and granules. Clypeus with disc covered with plastron setae; anterior and lateral areas without plastron setae, covered with sparse, long setae. Labrum transverse, narrower than clypeus, lateral margins with long bristles. Antenna (Fig. 1D) 7-segmented with apical antennomere clubbed.

Pronotum (Fig. 3B) slightly wider than long, widest at basal 2/5, gradually narrowed from basal 2/5 to apex. Surface finely punctate; covered with sparse, long setae and with plastron setae laterally. Median longitudinal sulcus distinct and long, extending from base nearly to anterior 1/4, with a pair of small round fovea near base. Sublateral grooves straight, short, less than 1/3 length of pronotum. Anterior margin distinctly curved, anterior angles not produced. Lateral margins gradually curved. Basal margin trisinuate, emarginate anterior to scutellum, posterior angles nearly orthogonal. Prosternal process (Fig. 3C) very wide, with apex broadly rounded; surface distinctly microreticulate, glabrous.

Scutellum (Fig. 3B) cordate, longer than wide, widest at basal 1/4; surface microreticulate and glabrous. Anterior margin strongly curved, lateral margins weakly curved, apex acutangular.

Elytra (Fig. 3F) about 1.5 times as long as wide, widest near in median 1/3. Strial punctures large in basal 2/3 of elytra, separated by about twice a diameter; much smaller and more widely separated in other parts of elytra. Elytral intervals 5, 6, 7 with granulate carinae extending from base of elytra nearly to apex. Interval 5 to lateral margins covered with plastron setae. Hing wings reduced.

Metaventrite (Fig. 3D) with disc distinctly microreticulate and covered with sparse, long setae; lateral areas with plastron setae. Median sulcus shallow and indistinct, less than half the length of metaventrite.

Admedian carinae of abdominal ventrite 1 indistinct, straight, extending from base to near apex. Median areas of ventrites 1–4 (Fig. 3E) and anteromedial area of ventrite 5 microreticulate, covered with sparse, long setae; remaining surface of ventrites 1–5 covered with plastron setae and mixed with sparse, long setae.

Legs simple, surface granulate (except tarsi). Surface of femora surface covered with plastron setae; inner side of tibia with cleaning fringes; tarsi slightly shorter than tibiae; tarsal claws simple.

Aedeagus (Fig. 4H–K) slender and elongate. Median lobe symmetrical, widest at base, weakly narrowed from base to apex; apex rounded; with two pairs of long, elongate sclerotizations located at basal 1/2; a very thin, very long sclerotization extends from middle of median lobe to well beyond the apex, where it is curved. Sternite IX (Fig. 4L) with apical margin weakly emarginate, without setae, median strut curved at middle with base curved.

Measurements: CL: 1.03–1.11 mm; PL: 0.33–0.35 mm, PW: 0.42–0.44 mm; EL: 0.70–0.78 mm, EW: 0.50–0.54 mm.

Female externally similar to the male, averaging larger. Ovipositor as in Fig. 4M, N: valvifer about twice as long as coxite, distinctly expanded at base; coxite apex strongly expanded, roundly broadened at outer margin; stylus short, weakly curved.

Measurements: CL: 1.04–1.17 mm; PL: 0.33–0.38 mm, PW: 0.39–0.44 mm; EL: 0.72–0.78 mm, EW: 0.49–0.57 mm.

#### Distribution.

China. Only known from the type locality in Luodian County, Qiannan Buyi and Miao Autonomous Prefecture, Guizhou Province.

**Figure 5.**
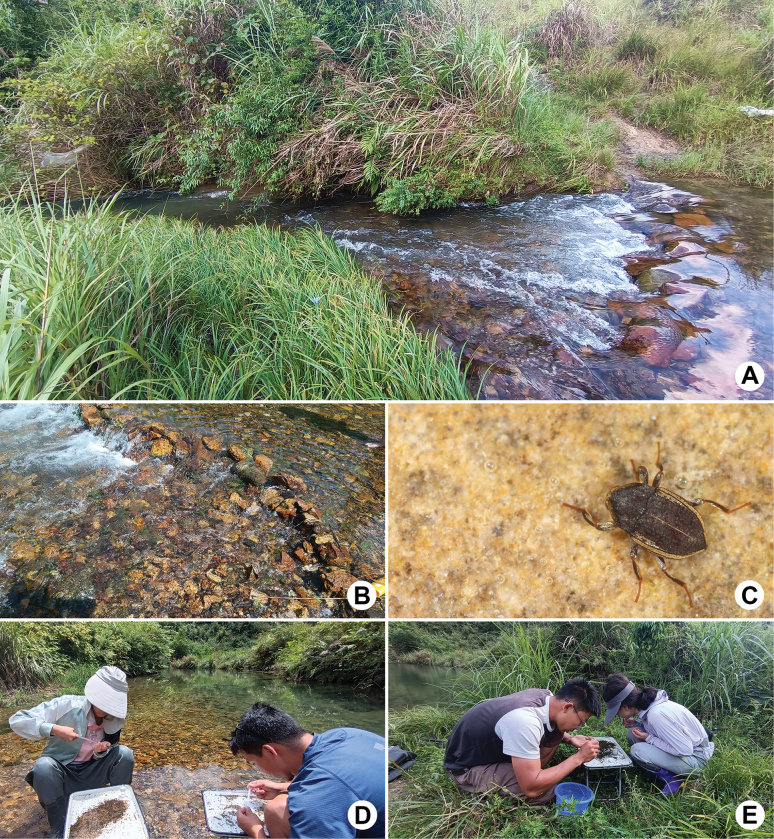
Habitat of *Sinonychuslipinae* sp. nov. at the type locality **A** general environment **B** microenvironment **C** living adult **D** collectors, left: Miss Yu-Hao Zhang, right: Dr Yin-Lin Mu **E** collectors, left: Dr Hai-Tao Li, right: Dr Pin Li.

#### Biology.

**Figure 6.**
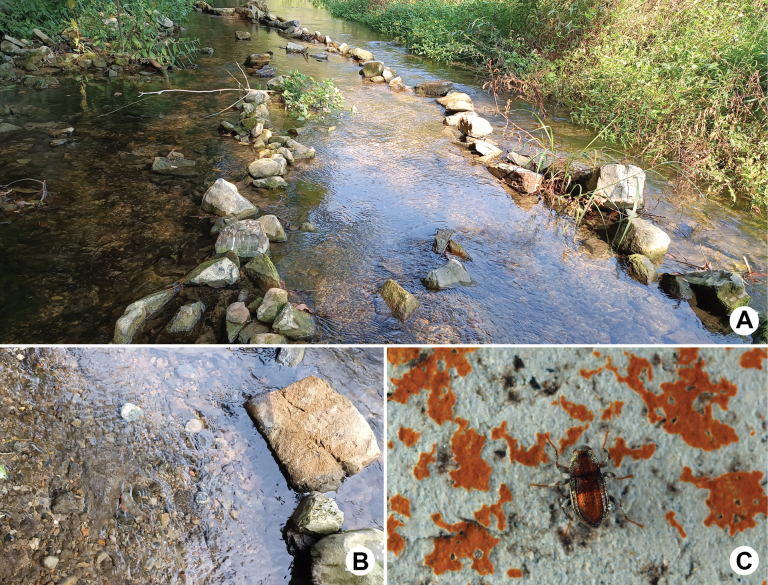
Habitat of *Sinonychusluodianensis* sp. nov. at the type locality **A** general environment **B** microenvironment **C** living adult.

All adults were collected from gravel on the bottom of a small stream in a ravine (Fig. 6A–C).

#### Etymology.

The specific epithet “*luodianensis*” refers to the type locality, Luodian County, Qiannan Buyi and Miao Autonomous Prefecture, Guizhou Province; the name is treated as an adjective.

#### Comparative diagnosis.

The new species can be easily distinguished from other *Sinonychus* species by its scaly plastron setae, and males by the aedeagus with very long, thread-like sclerotization extending from the middle of the median lobe and exceeding its apex.

### ﻿Key to known species of the genus *Sinonychus* Jäch & Boukal, 1995

**Table:** 

1	Plastron setae scaly, median lobe of aedeagus with very long sclerotization extending from middle of median lobe and extending beyond its apex	***S.luodianensis* sp. nov. (China)**
–	Plastron setae spiculate, sclerotizations of median lobe not as above	**2**
2	Elytral intervals 3 and 5–7 with granulate carinae	**3**
–	Elytral intervals 3 without granulate carina	**4**
3	Basal 1/2 of median longitudinal sulcus distinctly wider than apical 1/2; median lobe of aedeagus about 6 times as long as phallobase, base of sclerotizations of median lobe near middle of median lobe	***S.lipinae* sp. nov. (China)**
–	Basal 1/2 of median longitudinal sulcus not distinctly wider than apical 1/2; median lobe of aedeagus about 5 times as long as phallobase, base of sclerotizations of median lobe behind middle of median lobe	***S.tsujunensis* Yoshitomi & Nakajima, 2012 (Japan)**
4	Mandible with two apical teeth; antennal segment 7 covered with short setae apically	***S.satoi* Yoshitomi & Nakajima, 2007 (Japan)**
–	Mandible with three apical teeth; antennal segment 7 covered with long setae apically	***S.lantau* Jäch & Boukal, 1995 (China)**

## ﻿Discussion

Members of the tribe Macronychini are characterized by the reduced antennomeres and parameres of the aedeagus (Jäch and Boukal 1995). Members of this tribe usually possess a well-developed internal sac of the aedeagus; sclerotized structures of aedeagus can be observed and are usually seen as important characters to distinguish different species (Jäch and Boukal 1995; Hayashi and Yoshitomi 2015).

*Sinonychusluodianensis* sp. nov. shows a very special sclerotized structure that is quite rare in the tribe Macronychini, even in the family Elmidae: a very long and thin thread-like sclerotization extending from the middle of the median lobe and exceeding the apex of the median lobe. The functional significance of this structure is unknown. In our observations, the special long sclerotization appears to have the same origin as other shorter sclerotizations of the median lobe.

On the other hand, *Sinonychusluodianensis* sp. nov. has scaly plastron setae, which are quite different from those of other *Sinonychus* species. However, this species can be placed in *Sinonychus* under the current definition of the genus, while its true identity remains somewhat of a conundrum. The same situation is also present in other Macronychini members: e.g., Bian, Hu and Tong (2024) described *Cuspideviapilosus* Bian, Hu & Tong, 2024, which could be placed in the genus *Cuspidevia*, but it also shows some unusual characters, such as the pronotum without any median impression. Those problems remind us that systematic work on the tribe Macronychini based on molecular data is still very incomplete.

## Supplementary Material

XML Treatment for Sinonychus

XML Treatment for Sinonychus lipinae

XML Treatment for Sinonychus luodianensis

## ﻿Additional information

### Data availability

All of the data that support the findings of this study are available in the main text.
